# The study design and methodology for the ARCHER study - adolescent rural cohort study of hormones, health, education, environments and relationships

**DOI:** 10.1186/1471-2431-12-143

**Published:** 2012-09-05

**Authors:** Katharine Steinbeck, Philip Hazell, Robert G Cumming, S Rachel Skinner, Rebecca Ivers, Robert Booy, Greg Fulcher, David J Handelsman, Andrew J Martin, Geoff Morgan, Jean Starling, Adrian Bauman, Margot L Rawsthorne, David L Bennett, Chin Moi Chow, Mary K Lam, Patrick Kelly, Ngiare J Brown, Karen Paxton, Catherine Hawke

**Affiliations:** 1 Academic Department of Adolescent Medicine, University of Sydney, at Children’s Hospital, Westmead, Sydney NSW 2145, Australia; 2 Thomas Walker Hospital (Rivendell) Child, Adolescent & Family Mental Health Services Hospital Rd Concord West, Sydney, NSW 2138, Australia; 3 School of Public Health, University of Sydney, Sydney, NSW 2006, Australia; 4 Discipline of Pediatrics and Child Health, University of Sydney at Children’s Hospital Westmead, Sydney, NSW 2145, Australia; 5 The George Institute for Global Health and School of Public Health, University of Sydney, Sydney, NSW 2006, Australia; 6 National Centre for Immunisation Research and Surveillance of Vaccine Preventable Disease, Kids Research Institute, The Children’s Hospital at Westmead. Sydney Institute for Emerging infections and Biosecurity (SEIB), The University of Sydney, Sydney, NSW 2006, Australia; 7 University of Sydney and Department of Endocrinology, Royal North Shore Hospital, Sydney, NSW 2065, Australia; 8 ANZAC Research Institute, University of Sydney and Andrology Department, Concord Hospital, Sydney, NSW 2006, Australia; 9 Faculty of Education and Social Work, University of Sydney, Sydney, NSW 2006, Australia; 10 Northern Rivers University Department of Rural Health, Medical School, University of Sydney, PO Box 3074, Lismore, NSW 2480, Australia; 11 Department of Psychological Medicine, The Children’s Hospital at Westmead, Locked Bag 4001, Westmead, NSW 2145, Australia; 12 Social Work & Policy Studies, Faculty of Education and Social Work, University of Sydney, Sydney, NSW 2006, Australia; 13 Department of Adolescent Medicine and NSW Centre for the Advancement of Adolescent Health, The Children’s Hospital at Westmead, Sydney, NSW 2145, Australia; 14 Discipline of Exercise and Sport Science, The University of Sydney, Lidcombe, NSW 2141, Australia; 15 Faculty of Health Sciences, The University of Sydney, Lidcombe, NSW 1825, Australia; 16 Professor of Indigenous Health & Education, University of Wollongong, Wollongong, NSW 2522, Australia; 17 School of Rural Health, University of Sydney, PO Box 1043, Dubbo, NSW 2830, Australia; 18 School of Rural Health, University of Sydney, PO Box 1191, Orange, NSW 2800, Australia

**Keywords:** Puberty, Hormones, Adolescent, Cohort studies, Rural health, Behaviour, Wellbeing, Public health, Protocol, Paediatrics

## Abstract

**Background:**

Adolescence is characterized by marked psychosocial, behavioural and biological changes and represents a critical life transition through which adult health and well-being are established. Substantial research confirms the role of psycho-social and environmental influences on this transition, but objective research examining the role of puberty hormones, testosterone in males and oestradiol in females (as biomarkers of puberty) on adolescent events is lacking. Neither has the tempo of puberty, the time from onset to completion of puberty within an individual been studied, nor the interaction between age of onset and tempo. This study has been designed to provide evidence on the relationship between reproductive hormones and the tempo of their rise to adult levels, and adolescent behaviour, health and wellbeing.

**Methods/Design:**

The ARCHER study is a multidisciplinary, prospective, longitudinal cohort study in 400 adolescents to be conducted in two centres in regional Australia in the State of New South Wales. The overall aim is to determine how changes over time in puberty hormones independently affect the study endpoints which describe universal and risk behaviours, mental health and physical status in adolescents. Recruitment will commence in school grades 5, 6 and 7 (10–12 years of age). Data collection includes participant and parent questionnaires, anthropometry, blood and urine collection and geocoding. Data analysis will include testing the reliability and validity of the chosen measures of puberty for subsequent statistical modeling to assess the impact over time of tempo and onset of puberty (and their interaction) and mean-level repeated measures analyses to explore for significant upward and downward shifts on target outcomes as a function of main effects.

**Discussion:**

The strengths of this study include enrollment starting in the earliest stages of puberty, the use of frequent urine samples in addition to annual blood samples to measure puberty hormones, and the simultaneous use of parental questionnaires.

## Background

Adolescence is a time when interventions have the capacity to make changes in individual health trajectories [1-3]. The aim of this study is to understand the effects of longitudinal changes in puberty hormones, especially the onset and tempo of change, on adolescent health and well-being. The present paper presents the research protocol for the study, with an emphasis on the newer technologies which have made this type of study possible.

**Figure 1:**
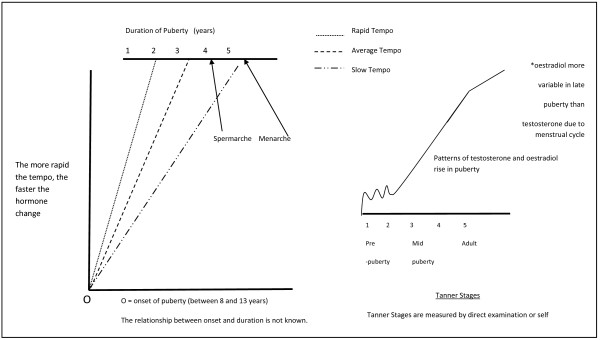
**Puberty hormone change and tempo.** Shows the pattern of testosterone and oestradiol change during puberty, the puberty milestones of menarche and Spermarche in relation to puberty stage and how the tempo of puberty may vary between individuals.

The two prime hormones, or biological measures, of puberty are testosterone (in boys) and oestradiol (in girls) [4], [Figure 1. The dramatic hormone changes of puberty are a universal and unforgettable experience. Inevitably such a phenomenon prompts many assumptions about the effects of puberty hormones on wellbeing and health. However, the authentic, longitudinal effects of the puberty hormones on human health and wellbeing in adolescence and in later adult life are not well understood, and remain under-researched. Previous research has relied on proxy measures of puberty hormonal changes including self-report of physical change and physical growth [5].

The major and rapid increases in puberty hormones mean that repeated hormone measurement is essential to appraise the relationships between biological variation in puberty hormones and the concomitant adolescent health problems and to allow exploration of the biological determinants of individual, social and physical environmental factors that are prominent in adolescence [6]. This is the first challenge in research of this nature. The onset of puberty occurs when testosterone and oestradiol begin to rise above the levels of childhood. This event occurs anywhere between the ages of 8 and 13 years [7,8] but chronological age is an unreliable indicator of pubertal stage. The tempo, or time, to complete the pubertal increase in reproductive hormones is comparatively rapid, between 18 months and four years, which is too rapid to be characterized by annual or less frequent blood sampling. Although the impact of variable tempo of puberty on health and wellbeing has not been well studied (or reported) [9], we propose that tempo is a key variable mediating the clinical effects of dynamically changing puberty hormones on adolescent health and behaviour [10]. It is notable that hormonal events of puberty are as dramatic in scope and amplitude as more well known examples of rapid hormonal change that cause defined physical, behavioural and mental effects such as the menstrual cycle, pregnancy, castration, anabolic steroid use and the menopause [11-13].

**Figure 2:**
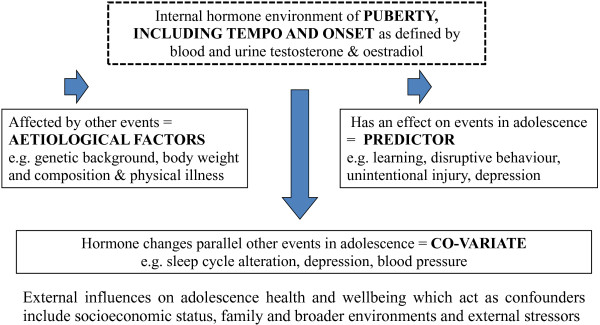
Depicts how the hormonal environment of puberty as defined by blood and urine testosterone and oestradiol may be related to the events of adolescence.

The additional challenges to research on puberty hormones are to create a framework where factors can be repeatedly studied with minimum reporter burden, and to ensure that the interactions between factors are understood and accounted for in order to satisfactorily interpret outcomes. The factors that have been chosen for study are those that are objectively measurable as well as being clinically important and modifiable, thus forming the basis of subsequent interventional studies. These factors are listed below and a brief review of the factors, how these are interrelated and their roles as possible aetiological factors, co-variates or predictors follows [Figure 2].

The Study Variables are:

Universal aspects of adolescent health and wellbeing which include social and emotional wellbeing, educational engagement and motivation, and sleep.

Health risk behaviours which include unintentional injury, alcohol and other drugs, sexual activity and conduct disorder.

Mental health which includes depression, anxiety and self harm.

Physical health which includes chronic illness and disability and cardiovascular risk factors – overweight, physical inactivity and tobacco use.

### Universal aspects of adolescent health and wellbeing

Socially competent adolescents are more likely to do well in education and less likely to have mental health problems [14]. Learning and engagement in education in adolescence is critical to social and emotional development and well-being, but the influence of the onset of and tempo of pubertal hormone rise on motivation and engagement in education, particularly when compared with the effects of socio-economic status and mental health, is as yet unknown [15]. Sleep disturbances in adolescents are common and may affect school performance and mood. The most common adolescent sleep disorder is delayed sleep phase syndrome (5-10%) [16]. What is not known is how the onset and tempo of pubertal hormone changes relate to sleep alterations and disturbances [17].

### Health risk behaviours

Unintentional injury is the most common cause of death in adolescents [18], mainly as a result of road traffic accidents [19], poisoning and drowning. Previous work has highlighted possible associations between pubertal stage as estimated by direct physical examination and physical injury incidence [20], but the onset of and the rate of the rise in pubertal hormones have yet to be studied in this context. Drug and alcohol use in adolescence increases the likelihood for reduced educational attainment, co-morbid mental health disorders, substance abuse and dependence, criminality, and psychiatric disorders in adulthood [21]. We propose that puberty onset and tempo also play an important role in these associations [22]. Sexual activity in teenagers is occurring at younger ages and teenagers are more likely to report multiple partners than in previous generations [23]. The relative contribution of changing pubertal hormone levels, specifically tempo, to sexual activity has not been confirmed and the mechanism of their influence and modifiers are completely unknown. Conduct disorders appear in pre-puberty or first emerge in puberty [24]. There are conflicting data about these disorders and their relationship to early or late maturation as measured by self report and single measurements of blood testosterone levels.

### Mental health

Depression and anxiety are the most common mental health problems in young people. At any time point, up to five percent of adolescents experience depression severe enough to warrant treatment, and around 20% of adolescents will have experienced significant depressive symptoms by adulthood [25,26]. On self reported Tanner Stages, depression occurs most commonly at Stage 3 in girls and before Stage 3 in boys. There is conflicting evidence about whether age of onset of puberty influences depression [27-29]. Higher rates of self-harm are found among adolescents in self-reported late puberty compared to early puberty [30]. The association is mediated by depressive symptoms, sexual activity and alcohol intake, and often attenuates with increasing age [31].

### Physical health

Fifteen percent of adolescents have a chronic physical illness or disability [32]. If serious this may retard the onset of puberty and potentially alter tempo [33]. Overweight, physical inactivity and tobacco use, which are the top three contributors to the burden of disease in developed countries [34], all increase during adolescence [35]. Twenty percent of adolescents smoke by the age of 17 years [36]. There are no longitudinal studies on how the age of onset and rate of change or tempo of puberty hormones influence the cardiovascular risk factors of overweight, physical inactivity and tobacco use.

## Methods/Design

### Introduction

The ARCHER study is a multidisciplinary, three year, longitudinal cohort study, using a convenience sample. The research hypothesis of the study is that onset and/or tempo of the rise in puberty hormones play a significant role in adolescent health and wellbeing. The ARCHER study commenced recruitment in June 2011 and has enrolled 200 adolescent participants (with parent/guardian) as of March 2012.

A feasibility study for all aspects of baseline data collection was completed in 28 students in years 5&6 at a rural primary school. Sample collection, labeling, transport and storage were successful. The plasma testosterone and oestradiol levels demonstrated that the participants (except 2 -1 M, 1 F) had levels consistent with pre- or very early puberty. On-line questionnaires were revised and successfully re-piloted in August 2009 in a rural primary school with 26 students and their parents. In 2010 focus group discussions (single and mixed gender with 10–12 year olds and 13–15 year olds) were held with 58 participants. Topics included what would positively influence recruitment and retention, how peers might participate in the study and the collection of biological samples. The young people expressed strong preferences in relation to the collection process and the focus group outcomes are now published [37]. The methodology was further tested in 2010 in a funded pilot study on puberty, sleep and depression, with included the use of the Actiwatch. This is a digitally integrated recording of wrist activity which provides more detailed information on sleep wake/times [38].

### Setting and study sample

The study is being conducted in Dubbo and Orange, two large towns in regional Australia in the State of New South Wales and surrounding areas. Enrollment is in school Grades 5, 6 and 7 (10–12 years of age) to ensure that both the transition into early puberty and the end of puberty are captured in adequate numbers. Each student will require a parent or guardian to also participate in the study. Lack of parental competence in English or adolescent intellectual disability are exclusion criteria. The least common primary outcome that we measure is depression, which we estimate will affect 5% of children in mid- to late-puberty [39]. We estimate that 160 boys and 160 girls at Tanner Stage 3 (the pubertal stage where clinical onset of depression is most likely) are required to detect a one standard deviation (SD) difference in testosterone nmol/l (SD = 5) [40] or oestradiol pmol/l (SD = 250) [41] levels between those with and without depression. The anticipated loss to follow up is 5% per annum in this setting. Assuming a mean age of puberty onset of 11.4 years [42], 400 subjects will ensure adequate numbers of adolescents at Tanner Stage 3 in the study, as a few younger subjects may not reach this Stage at the end of the study (age 14 years) and some older children may have already entered Stage 3 at the beginning of the study (age 12 years).

### Recruitment procedures and follow up rates

Recruitment is primarily through schools, as well as through the media (targeted at parents) and through established links with local community groups. We have permission from the state Department of Education and Communities - Western Region and the Catholic Education Office – Bathurst Diocese to recruit through their schools. Data collection does not take place in the schools. Information sessions are held in the community and at consenting schools. Parents are asked to provide two additional adult contact names with the written consent, to reduce loss to follow up. Participants who agree are sent birthday cards, updates and study newsletters as reminders by their preferred mode of contact (email, letter or Short Message Service (SMS)). These practices have been shown to increase retention rates in school age children [43]. The young people requested ‘get togethers’ in the focus groups to celebrate their involvement in the study and these will be held annually.

### Data collection

Adolescents will complete questionnaires at baseline and annually for the following three years. We will use a computerized on-line questionnaire with branched algorithmic structure, to enhance confidentiality and accuracy of responses and to reduce exposure to sensitive questions (particularly related to sexual activity). This has been created and trialed. The total number of questions for adolescents is 256. These are completed in approximately one hour (shorter for the older participants and closer to 75 minutes for younger participants). Adolescents complete the questionnaire under supervision of research staff. Questionnaires will not be performed in the fasted state and, as with all other data and biological sample collection, are done outside school hours. Physical examination occurs at baseline and annually for three years. Blood is collected at baseline and annually for three years, in the fasting state. A first morning urine sample is collected at baseline and every three months for three years. A single indicator question providing information on mood fluctuation is sent to all participants with access to mobiles phones every 3 months and functions as a reminder to complete urine collection.

A parent or guardian completes a questionnaire at baseline and annually for three years, and in the same month as their child. The questionnaires are available online or as hard copy and contain approximately 180 questions. This questionnaire reduces reporter burden on adolescents for demographic data, as well as providing other relevant family and environmental data.

### Ethics

Ethical approval has been granted for the pilot questionnaires, anthropometry, biological sample collection (HREC 10612), the pilot SMS and sleep studies (HREC 12502) and for the full study described in this protocol (HREC 13094) from the Human Research Ethics Committee, University of Sydney.

### Instruments

#### Questionnaires - adolescent

The Child Behaviour Check List (CBCL) in the version validated for ages 11–18 years as the Youth Self Report (YSR) [44] is the main questionnaire measurement instrument. It contains 20 social competence items that measure participation in hobbies, games, sports, jobs, chores, friendship, and activities and 8 sub-scales, measuring internalizing and externalizing behaviour. In addition the subscales provide data on social competence, learning and engagement in education, sleep, unintentional injury, drug and alcohol use, conduct disorders, disruptive behaviour disorders, depression and anxiety, and self harm. The YSR also identifies the presence of physical illness and disability.

The YSR is supplemented by the following questionnaires.

a) Selected measures of self competency taken from the Raine cohort [45], including Cowen’s Self Efficacy [46] and Adolescent Self Perception Profile [47].

b) 12 selected items from Motivation and Engagement; Enjoyment of School, Academic Buoyancy and Class Participation scales [48,49]. These results can be linked to the Australian National Assessment Program Literacy and Numeracy scores [50].

c) A questionnaire is used to document sleep/wake patterns, and sleep disorders in the adolescent’s natural sleeping environment [51]. In addition, a convenience subset of adolescents will use the Actiwatch home monitor system annually.

d) Direct questions on unintentional injury type in the preceding 12 months provided by RI, selected questions from The Australian School Students Drug & Alcohol Survey [52], branched age appropriate sexuality questions on romantic relationships, sexual feelings, history of STI and pregnancy [23] and the 40 item validated Australian self-reported delinquency scale by Mak [53] in those adolescents who score highly on the YSR subscale for externalizing problems.

e) The Short Moods and Feeling Questionnaire (13 item) in all subjects [54]. The short form has been validated as a self-reported unidimensional measure of symptom severity of childhood depression in community samples.

f) The 16 item Deliberate Self Harm inventory [55] for those who respond with a 1 or 2 for YSR item 18 or 91 (relating to self-harm or suicide).

g) Selected questions from Health Behaviors in School-aged Children for physical inactivity, nutrition and tobacco [56].

h) One SMS question which is a linear analogue self-assessment scale question on mood providing information on mood fluctuation [57].

i) Tanner Stage of puberty by self report using standardized line drawings which allow determination of accuracy of this method of pubertal staging, which is the main method used in epidemiological and non-clinical studies.

#### Questionnaires – parent/guardian

a) Demographic data for family and adolescent.

b) Child Behavior Checklist (CBCL) validated questionnaire for parents of children aged 6–18 years to corroborate the adolescent’s YSR [58].

c) The validated Macmaster’s Family Assessment Device [59] to obtain information on family and local environmental factors, which are potential confounders.

After the final 3-year questionnaire all adolescents will be asked whether they believed that the speed of their puberty was faster, the same as or slower than their age peers and whether the onset of their puberty was earlier, the same as or later compared to their age peers. Their parent/guardian will be asked the same questions in relation to their child.

#### Physical examination – adolescents only

Weight and body composition is measured in light clothing using Tanita TBF-300 Pro Body Composition Analyzer [60]. Height, and foot length as an indicator of growth stage [61] are measured on a portable stadiometer (to 0.1 cm) and with a metal ruler (to 0.5 cm) respectively, and waist [62] with a tape (to 0.1 cm) using standard techniques. Body Mass Index, as a measure of overweight (kg/m^2^) and waist circumference are expressed as individual z-scores based upon age and sex related reference charts [63]. Blood pressure (BP) and pulse rate are measured using an automated BP monitor, under standardised conditions.

#### Laboratory – adolescents only

Fasting bloods are collected using local anaesthetic cream and separated serum and spot urine samples frozen at −80°C, as well as buffy coat in one sample. Total blood draw is approximately 30 ml. Menstrual cycle stage will be recorded in post-menarchal females, with the perimenstrual week avoided.

### Blood

Testosterone and oestradiol will be measured using AP5000 LC Tandem MS in the Andrology laboratory, ANZAC Research Institute [64] (DHEA, DHT and oestrone will also be measured). There will be adequate blood collected to measure other potential biological variables of interest, which will form the basis of future studies but which are not integral to this study. These variables include LH, FSH, SHBG and IGF-1, inhibin, anti-Mullerian hormone, ACTH, cortisol, growth hormone, prolactin, oxytocin, TSH, thyroxine, triiodothyronine and Vitamin D (all of which are hormones relevant to puberty) [4], full blood count, ferritin, glucose, liver function, urea, electrolytes, full lipid profile, creatinine, and insulin, fatty acid profile, leptin, adiponectin, resistin, TNF alpha, CRP and Interleukin 6 (as indicators of cardiovascular risk and also to derive eGFR) [65]. There is the potential to be able to study genes relevant to puberty and sex steroid action; KiSS-1, KAL1, FGFR1, GnRHR, GRPR-54, TAC3, neurokininB, androgen and oestrogen receptor, steroidogenic enzymes such as CYP, StAR, P450scc, P450 (17alpha) and 17beta-HSD [66], and genes relevant to body composition and insulin resistance, such as leptin gene and product, MC4R, FTO, IL-6, and UCP-2 [67].

### Urine

A first morning, fasting urine sample will be collected at home. Urinary testosterone and oestradiol will be measured, also using AP5000 LC Tandem MS. Urinary hormones, although less well standardized than blood, are essential to define hormone trajectories which can never be accomplished by a single annual sample. Sperm (indicating spermarche) will be measured in male urine samples [68]. There will be adequate urine to measure creatinine and microalbumin, but these measures are not integral to the current study.

#### Geocoding

Residential address will be geocoded [69] to obtain additional information such as neighbourhood socioeconomic status, access to services and amenities and other environmental exposures, which may be study confounders for which adjustment will be required.

### Statistical analysis

The main independent variables are tempo of puberty and age of onset at puberty. Both will be measured by testosterone and oestradiol, in blood and urine. Data analysis will have three main components.

1) The first component is testing the reliability and validity of measures under focus. These analyses will centre on: (a) descriptive, reliability and item functioning; (b) factor analysis to test factor structure and validity of measures; (c) ANOVAs for preliminary tests of main and interaction effects of background socio-demographics; and (d) tests of (in)variance in factor structure to ensure congruence in measurement properties across sub-groups to justify pooling sample data for subsequent modeling throughout the project [70].

2) The second component is a correlational one and assesses the impact of tempo and onset of puberty over time. Specifically, for example, by assessing in the one analytic model (eg. in linear regression models for continuous variables such as YSR scores for sensation seeking and in logistic regression models for dichotomous outcomes such as depression or sexual debut) the effects of Time 1 onset and tempo of puberty on Time 2 outcomes after controlling for Time 1 outcomes, it is possible to get a sense of the relative salience of the onset, and tempo of puberty in one time period over factors in a following time period [71]. Also in these longitudinal analyses, key socio-demographic (and other) factors such as gender, school grade and socioeconomic factors can be included to get a further sense of puberty by controlling for other potential influences. The three monthly urine samples for the biological markers of puberty can be used to regress time on time measures, with tempo being defined by the residuals. Thus the larger and the more positive a residual over time, the more rapid the tempo of change.

3) The third component is based on mean-level differences (e.g. repeated measures) and explores for significant upward and downward shifts in means on target outcomes as a function of main effects (e.g. early and late onset; rapid and slow tempo) and interactions (e.g. early onset/rapid tempo; late onset/rapid tempo; early onset/slow tempo and late onset/slow tempo) of puberty effects that also control for key socio-demographic (and other) factors as covariates [72]. Taken together, these analyses (and adaptations of them) allow for integrative tests of the substantive and methodological issues at hand.

Analyses will be conducted using SAS, Stata and WinBUGS (for repeated measures analyses).

## Discussion

This study aims to extend previous puberty research which has sought to consider the relationship between the biological events of puberty and the complex, longer state of adolescence and its associated health issues. As a longitudinal study it commences at a younger age in order to capture the transition into puberty, as defined by the first elevations of testosterone and oestradiol in blood and urine. Thus puberty is defined using biological markers, which are of fundamental importance to the project. Previous studies have relied on self report of pubertal stage, which is a lagging and insensitive measure, that correlates only weakly with measured puberty hormones [5]. This will be the first study to focus on the biological tempo of puberty and its impact on adolescent health and wellbeing, with frequent biological sampling and the introduction of new, ultrasensitive state-of-the-art, mass spectrometric steroid assays. Previous studies have used immunoassay with unextracted serum, which has proven analytically inaccurate and unreliable (although faster) and which further places in doubt previously described relationships between the clinical features of puberty and its hormonal determinants.

The utility of this new knowledge, by the translation of hormone data into real life, is to better direct interventions at a time when developmental trajectories remain plastic and capable of alteration and without the need for biological sample collection. It can be done because ‘puberty phenotypes’ such as early or late onset (out of step with peers) or rapid tempo (rapid physical change) and which deviate most from average age of onset/average tempo (similar to the majority) are already recognizable using simple observation by clinicians, parents, educators and adolescents. What is missing is an understanding of the direction and magnitude of influence of intrinsic tempo and timing of puberty on adolescent behaviour, wellbeing and health and how hormonal changes interact with external factors. The indirect, non-biological evidence is that the magnitude of intrinsic hormonal influence is significant.

There is no suggestion that altering the onset or tempo of puberty is a realistic intervention (despite its feasibility using modern steroidal or gonadotrophin releasing hormone analogue therapy [73,74]). It is however of great interest to determine whether the dynamic hormonal changes of puberty trigger behavioural or physical problems. If puberty hormones do have an effect, it would be possible to target established, evidence-based directed interventions to at-risk adolescents, presumably those whose puberty patterns are dissimilar to the majority. These could include evidence-based interventions in sleep hygiene, unintentional injury, mental health, and cardiovascular risk. In schools, where year levels are defined by age, rather than pubertal stage, information will be provided to better assist school personnel to identify and deal with the wide developmental range in any given year group. Adolescents, their parents and families will benefit from understanding how hormones influence adolescent behaviours. The community will benefit if knowledge gained from this study is used to improve adolescents’ health and wellbeing.

## Competing interests

The authors declare that they have no competing interests.

## Authors’ contributions

KS is the study leader and has made a substantial contribution to the conception and design of the study and to the carrying out of the pilot and feasibility studies and focus groups which have informed final ARCHER study design. PH has made a substantial contribution to the conception and design of the study and has supervised a pilot study on sleep, depression and puberty. RC has contributed substantially to the study design, in particular expertise on power analysis and statistical analysis. RS has contributed to the overall study design, writing of the protocol in particular relating to the measurement of sexual and reproductive behaviour and risk-taking. She has also contributed to the design and interpretation of the pilot studies. RI has contributed to study design and questionnaire development, particularly relating to risk taking behaviours and injury. RB has contributed to study design, particularly on epidemiology and infectious diseases. GF has contributed to study design, in particular to the assessment of metabolic status. DH contributed to the study design and his laboratory is providing analyses of puberty hormone by mass spectrometry, including the development of urine assays. AM has contributed to the methodology for learning and engagement in education and to statistical analysis considerations. GM has contributed to study design in relation to geo-coding. JS has contributed to the choice of questionnaires for the assessment of mental health and behaviour. AB has contributed to sampling, cohort design and adolescent self-report measures. MR has contributed to study design, particularly in relation to social competency and designed and helped to carry out the focus group studies. DLB has contributed to recruitment and community engagement protocols. CMC has contributed to the assessment of sleep measures in adolescence. ML has contributed to the development of on-line survey instruments for young people and parental survey, and to the creation of the study database. PK has contributed to the study design around proposed analysis, including how to describe the biological puberty trajectories. NB has provided extensive expertise on the engagement, recruitment and study of indigenous participants. KP has been the project manager for the study from the beginning of the pilot work and has developed the questionnaire package and data collection protocols. CH has made a substantial contribution to the conception and design of the study and has supervised the pilot and feasibility studies and the community consultation. All authors read and approved the final manuscript.

## Authors’ information

KS (MBBS PhD FRACP) holds the Medical Foundation Chair in Adolescent Medicine at the University of Sydney Australia and is an adolescent endocrinologist. PH (BMedSc MBChB PhD FRANZCP) is Conjoint Professor of Child & Adolescent Psychiatry, Discipline of Psychiatry, Sydney Medical School, University of Sydney and Director, Infant Child & Adolescent Mental Health Services Sydney South West Area Health Service. RC (MB BS MPH PhD) is Professor of Epidemiology Sydney School of Public Health, University of Sydney. SRS (MBBS PhD FRACP) is a clinical academic and Associate Professor in the Discipline of Paediatrics and Child Health, University of Sydney and Adolescent Physician at the Children’s Hospital Westmead, Sydney Australia. RI (MPH PhD) is Director of the Injury Division at the George Institute for Global Health and Professor in the Sydney School of Public Health, University of Sydney. RB (MBBS (Hons) MSc MD FRACP FRCPCH) is Head, Clinical Research, The National Centre for Immunisation Research and Surveillance. GF (MBBS MD FRACP) is Director of Diabetes and Endocrinology at the RNSH Sydney, Chair of the Diabetes Network of NSCCAHS and Clinical Professor of Medicine at the University of Sydney. DH (MBBS PhD FRACP) is Professor of Reproductive Endocrinology & Andrology. AM (BA(Hons) MEd(Hons) PhD) is Professorial Research Fellow and Australian Research Council Future Fellow Faculty of Education and Social Work, University of Sydney. GM (PhD) is Associate Professor and Deputy Director of the Northern Rivers University Department of Rural Health and an Environmental Epidemiologist. JS is a Child and Adolescent Psychiatrist at Thomas Walker Hospital (Rivendell) Child, Adolescent & Family Mental Health Services and a Clinical Senior Lecturer, Discipline of Psychiatry, Sydney Medical School. AB (MBBS MPH PhD FAFPHM) is Professor, School of Public Health, University of Sydney. MR (PhD) is a Senior Lecturer, Social Work & Policy Studies at the University of Sydney Australia and is sociologist who research includes the experiences of rural young people. DLB (MBBS FRACP FSAHM), an adolescent health physician, is Clinical Professor of Adolescent Medicine at the University of Sydney, senior staff specialist with the Department of Adolescent Medicine and Head of the NSW Centre for the Advancement of Adolescent Health, The Children’s Hospital at Westmead. CMC (PhD) is a sleep scientist in the Faculty of Health Sciences, University of Sydney. ML (PhD) is an informatician in the Faculty of Health Sciences, University of Sydney. PK (PhD) is a Senior Lecturer (Biostatistics) in the School of Public Health at the University of Sydney. NB (BMed, MPHTM, RACGP) is a Professor of Indigenous Health & Education. University of Wollongong. KP (BHSc) is a registered nurse, the project manager of the ARCHER study and co-ordinated the pilot and feasibility studies. CH (MBBS FFPH) is a Senior lecturer in Rural Health at the School of Rural Health, University of Sydney and a public health physician.

## Pre-publication history

The pre-publication history for this paper can be accessed here:

http://www.biomedcentral.com/1471-2431/12/143/prepub
